# Baicalin attenuates monocrotaline-induced pulmonary hypertension through bone morphogenetic protein signaling pathway

**DOI:** 10.18632/oncotarget.18825

**Published:** 2017-06-28

**Authors:** Zhaohua Zhang, Luan Zhang, Chao Sun, Feng Kong, Jue Wang, Qian Xin, Wen Jiang, Kaili Li, Ou Chen, Yun Luan

**Affiliations:** ^1^ Department of Pediatrics, The Second Hospital of Shandong University, Jinan, China; ^2^ Central Research Laboratory, The Second Hospital of Shandong University, Jinan, China

**Keywords:** baicalin, pulmonary hypertension, vascular remodeling, NF-κB, BMP

## Abstract

Baicalin, a flavonoid compound extracted from roots of *Scutellaria baicalensis* Georgi (huang qin), it has been shown to effectively attenuates pulmonary hypertension (PH), however, the potential mechanism remains unexplored. In this study, we investigated the potential mechanism of baicalin on monocrotaline (MCT)-induced PH in rats. The results showed that baicalin attenuated lung damage in PH rat model through inhibiting the pulmonary arterial smooth muscle cell proliferation and induction of cells apoptosis. Furthermore, we demonstrated that baicalin inhibition the expression of nuclear factor-κB (NF-κB) p65 and bone morphogenetic protein (BMP) antagonists gremlin-1, but increased the expression of inhibitor of NF-κB (I-κBα), BMPR2, BMP-4, BMP-9 and Smad1/5/8. Additionally, baicalin suppression endothelial-to-mesenchymal transition in PH lung tissue. Collectively, we confirmed that baicalin via inhibition of NF-κB signaling to further activation of BMP signaling to have a therapeutic effect on PH and providing a promising therapeutic strategy for PH.

## INTRODUCTION

Pulmonary hypertension (PH) is a lethal syndrome characterized by pulmonary vascular obstruction caused, a sustained elevation of pulmonary pulmonary vascular resistance, vascular remodeling, right ventricular hypertrophy and failure [1, 2]. The pathogenesis of PH including pulmonary arterial endothelial cell dysfunction and pulmonary arterial smooth muscle cell (PASMCs) proliferation [3]. The neointimal formation and hyperplasia of the medial vascular wall due to an imbalance between proliferation and apoptosis of PASMCs [4, 5]. Although there are a large number of treatment options for PH, including inhaled nitric oxide, vasodilators, calcium channel blockers, intravenous prostacyclin, and endothelin receptor antagonists, most patients eventually become resistant to therapy and succumb to the disease. Therefore, novel approaches are urgently required for the treatment of PH.

Recent studies revealed that the disorder of bone morphogenetic proteins (BMPs) signaling deregulated the cell growth and differentiation, and contributed to pulmonary artery remodeling in the process of PH [6, 7]. BMPs are multifunctional proteins that regulate cells proliferation, differentiation, and apoptosis, which bind and activate heteromeric complexes of type I and type II receptors. BMPs and there receptors plays a major role in pulmonary hypertension. Type II receptors (BMPR2) significantly decreased in patients with primary PH and in animal models induced by monocrotaline, chronic hypoxia or transgenic mice [8, 9]. BMP4 could regulate the complex receptor signaling pathways associated with BMPRII in distal PASMCs during the process of pulmonary arterial remodeling in PH [10]. BMP9 not only protected pulmonary arterial endothelial cells from apoptosis and promotes vascular stability, but also increased BMPR2 gene expression [11].

Baicalin, a flavonoid compound purified from the dry roots of *Scutellaria baicalensis Georgi* (huang qin), has several biological effects [12–15]. Moreover, baicalin has therapeutic potential for the treatment of PH through inhibition pulmonary artery pressure and pulmonary vascular remodeling via anti-inflammatory response [12, 16]. In recent years, it has been shown that baicalin can inhibit PAMSCs proliferation and promote apoptosis [17, 18]. However, the potential mechanism remains unexplored. Here, we investigated the protective role and revealed the underlying mechanism of baicalin against lung damage in MCT-induced PH rat.

## RESULT

### Baicalin attenuates monocrotaline-induced pulmonary hypertension and pulmonary vascular remodeling in rats

**Figure 1:**
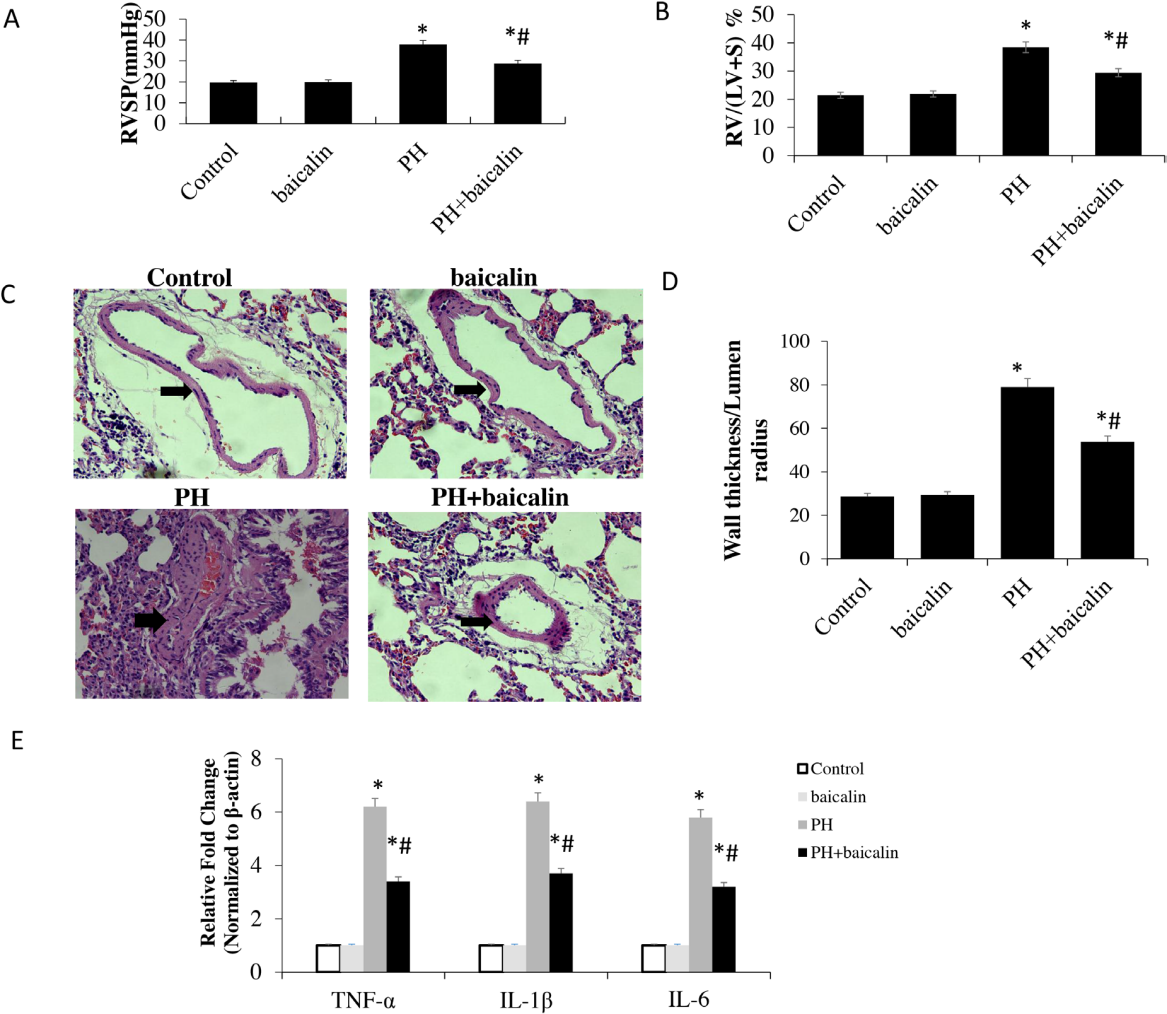
Effect of baicalin on MCT-induced pulmonary hypertension and expression of proinflammatory cytokine genes **(A)** A comparison of the right ventricular systolic pressure (RVSP) in each group. **(B)** A comparison of the ratio of right ventricular weight to left ventricle plus septum (RV/LV + S) in in each group. **(C)** Representative hematoxylin and eosin staining images in each group. **(D)** A comparison of the medial thickness of the pulmonary arterial walls in each group. **(E)** mRNA expression levels of tumor necrosis factor-α, interleukin (IL)-6, and IL-1β detected using quantitative real-time polymerase chain reaction. *P<0.05 *vs*. control; ^#^*P*<0.05 *vs*. PH group.

The ventricular systolic pressure (RVSP), right ventricle/left ventricle plus septum (RV/LV + S) ratio and pulmonary arterial pathological were used to evaluate the model of MCT-induced PH. The results showed that there were significant decrease in RVSP and RV/LV + S in PH + baicalin rats than MCT-induced PH rats (*P*<0.05; Figure 1A and 1B). To evaluate pulmonary vascular remodeling, the medial thickness of the pulmonary arterial was detected, H&E staining showed a significant decrease in the thickness of the pulmonary vascular walls in the smooth muscle layer of pulmonary arterioles in PH + baicalin rats than in PH rats (*P*<0.05; Figure 1C and 1D).

### Anti-inflammatory response of baicalin in rat lung tissue

The expression of proinflammatory cytokine / chemokine genes tumor necrosis factor-α (TNF-α), the interleukin (IL)-6 and IL-1β were detected by quantitative real-time polymerase chain reaction (qRT-PCR). Our results showed that the mRNA expression of TNF-α, IL-6, and IL-1β was increased in lung in MCT-induced rats than control, however, when the rats were treatment with baicalin, the mRNA expression of TNF-α, IL-6, and IL-1β was significantly reduced when compared with PH rats (*P*<0.05; Figure 1E).

### Effects of baicalin on BMP-related signaling molecules expression in the lungs

**Figure 2:**
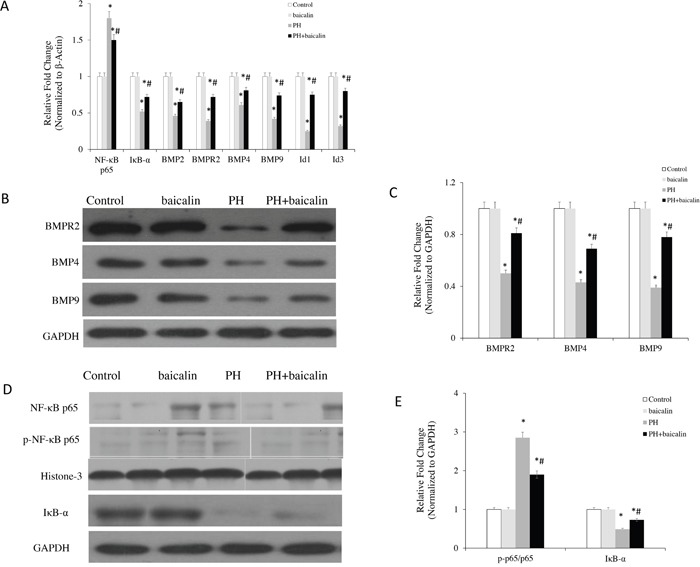
Effected of baicalin on NF-κB/BMP-related signaling molecules expression in the lungs **(A)** The mRNA level of NF-κB p65, IκB-α, BMPR2, BMP4, BMP9 in each group by quantitative real-time PCR analysis. **(B)** Western blots showing protein expression of BMPR2, BMP4, BMP9 in each group. **(C)** Normalized band intensity quantification showing the fold change of the BMPR2, BMP4 and BMP9. **(D)** Western blots showing protein expression of IκB-α, NF-κB p65 and p-NF-κBp65 in each group. **(E)** Normalized band intensity quantification showing the fold change of the IκB-α, NF-κB p65 and p-NF-κBp65. *P<0.05 *vs*. control; ^#^*P*<0.05 *vs*. PH group.

To determine the alteration of cell signaling molecules contributed to the baicalin in MCT-induced PH rat, the expression of NF-κB-BMP signaling axis molecules were determined. Our results showed that when the rats were treatment with baicalin, the mRNA expression of IκB-α, BMPR2, BMPs-2/4/9, Id1 and Id3 were significantly increased, but the mRNA expression of NF-κB p65 was decreased in lung than in MCT-PH rats (*P*<0.05, Figure 2A). Mover over, western blot results showed that the protein expression of IκB-α, BMPR2, BMPs-4/9 were up-regulation, but the protein expression of NF-κB p65, p-NF-κB p65 and the ratio between phospho and total NF-kB p65 (p-p65/p65) were down-regulation in baicalin + PH group than in PH group (*P*<0.05, Figure 2B, 2C, 2D and 2E).

**Figure 3:**
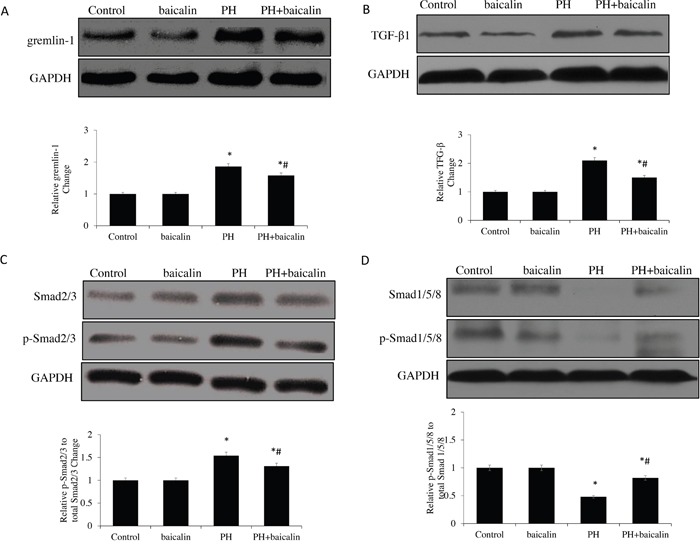
Effected of baicalin on protein expression of TGF-β1/Smads-related in lung **(A)** Western blots showing protein expression of TGF-β1. **(B)** Representative protein expression of gremlin-1. **(C)** Representative protein expression of Smad2/3 and p-Smad2/3. **(D)** Representative protein expression of Smad1/5/8 and p-Smad 1/5/8 *P<0.05 *vs*. control; ^#^*P*<0.05 *vs*. PH group.

The BMP antagonist, gremlin 1 was also measured in lungs, there was a significantly down-regulation of gremlin 1 protein expression in PH + baicalin lung compared with PH group (*P*<0.05, Figure 3A). Furthermore, the BMP related TGF-β1/Smad signaling molecules were also detected, the present study showed that the protein expression levels of TGF-β1 and the ratio of phospho-Smad2 to total Smad2 were significantly reduced, but the ratio of phospho-Smad 1/5/8 to total Smad 1/5/8 was significantly increased in the lung when the PH rats were treatment with baicalin than PH rats, which is consistent with reduced BMP signaling (*P*<0.05, Figure 3B, 3C and 3D).

### Apoptotic effects of baicalin *in vivo* and *vitro*

**Figure 4:**
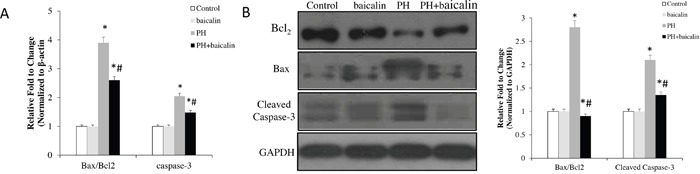
Effected of baicalin expression of apoptotic family genes in lung **(A)** Quantitative real-time PCR analysis of the mRNA expression of Bax/Bcl2 ratio and the level of cleaved caspase-3. **(B)** Western blots showing protein expression of Bcl2, Bax and cleaved caspase-3. Normalized band intensity quantification showing Bax/Bcl2 ratio and the level of cleaved caspase-3. *P<0.05, vs. control; ^#^*P*<0.05, vs. PH group.

RT-PCR results showed that the mRNA expression of antiapoptotic gene *Bcl2* was up-regulated in the PH rats, the proapoptotic genes *caspase-3* and the ratio of *Bax*/*Bcl2* were down-regulation when the MCT-PH rats were treatment with baicalin(*P*<0.05, Figure 4A). Western blot results showed that the expression of protein levels of Bcl-2, Bax and caspase-3 were consistent with mRNA expression (*P*<0.05, Figure 4B).

**Figure 5:**
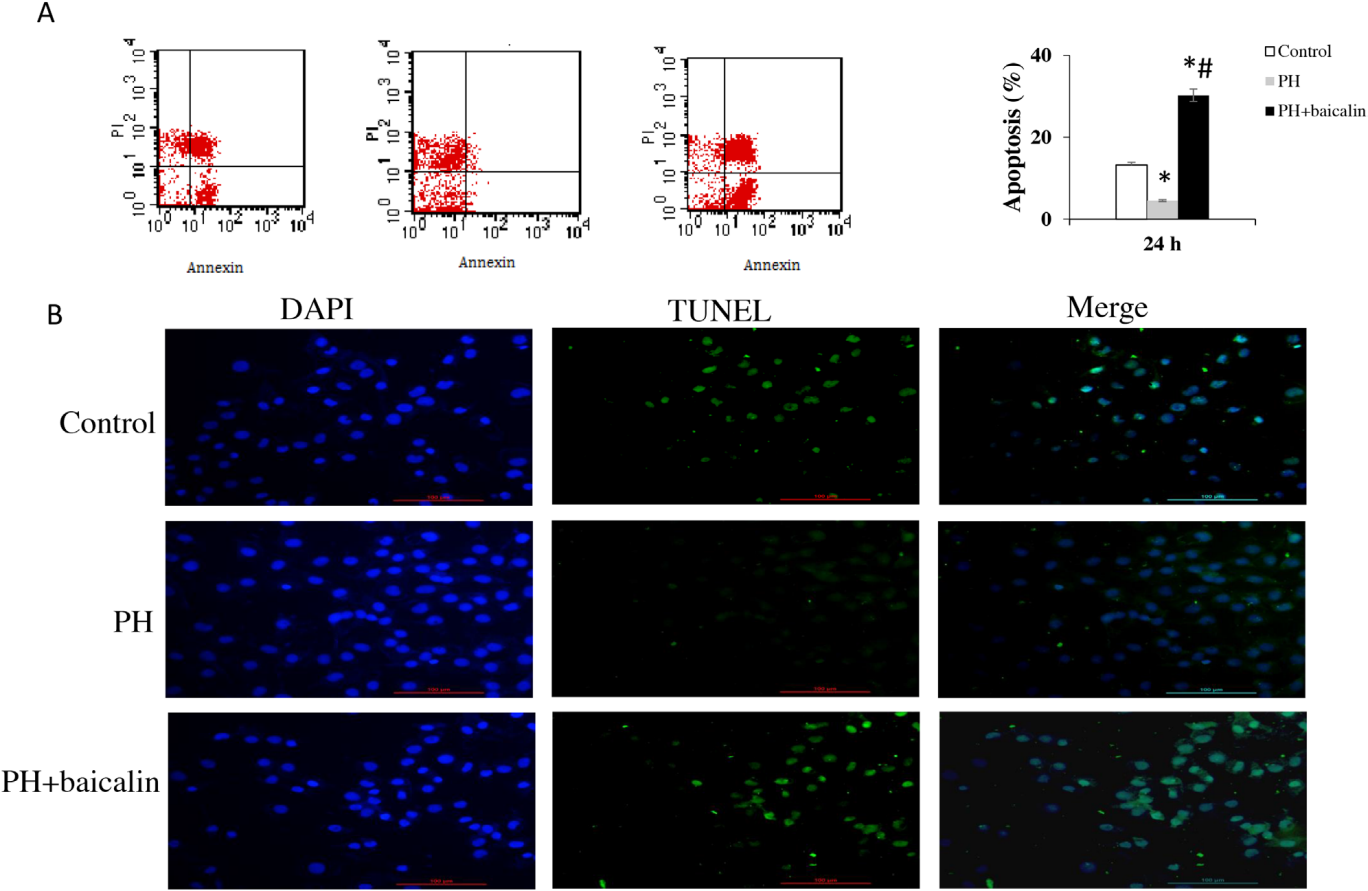
Effected of baicalin on apopotosis **(A)** Apoptosis of pulmonary arterial smooth muscle cell was determined by flow cytometry staining for Annexin V/FITC. **(B)** Cell apoptosis detection by TUNEL staining. *P<0.05, *vs*. control; ^#^*P*<0.05, *vs*. PH group.

Mover over, apoptosis *in vivo* were determined by flow cytometry staining with Annexin V/FITC and a One Step TUNEL apoptosis assay kit (Beyotime Institute of Biotechnology, Shanghai, China) according to the protocol. In the control group, the median percentage of apoptotic PAMSCs was 13.21%, MCT treatment in the rats resulted in increased percentage of apoptotic PAMSCs to 4.51% (*P*<0.01). The baicalin showed a significant reduction of apoptotic PAMSCs to 30.25%, compared with the MCT-treated PH group (*P*<0.05, Figure 5A).

The morphologic changes in PAMSCs by TUNEL staining showed that there was a significantly decreased of apoptosis in PH + baicalin group when compared with PH group (*P*<0.01, Figure 5B).

### Antiproliferative effects of baicalin *in vivo* and *vitro*

**Figure 6:**
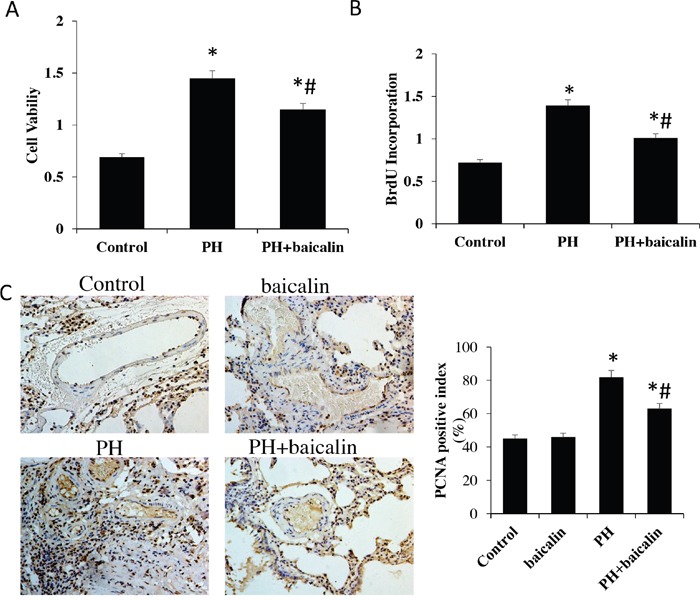
Effected of baicalin on pulmonary arterial hypertension smooth muscle cell proliferation **(A)** Cell viability was determined by MTT. **(B)** Population of cells was analyzed using a DNA BrdU incorporation assay. **(C)** PCNA in lung was detected by immunohistochemistry. *P<0.05, *vs*.control; ^#^*P*<0.05, *vs*. PH group. PCNA: proliferating cell nuclear antigen.

Cell viability and population were analyzed to demonstrate the effect of baicalin on PASMCs proliferation *in vivo*. Our results showed that the cell viability was increased and the population was decreased in MCT PH group compared with the control group, however, these change were obviously inhibited in PH + baicalin group when compared with PH group (*P*<0.05, Figure 6A and 6B).

Moverover, the number of PCNA as a marker of cell proliferation was detected *in vitro*. Immunohistochemistry of rat lung sections suggested that the number of PCNA positive cells in hypertrophied arteries was obviously reduced in PH + baicalin group conpared with PH group (Figure 6C).

### Effects of baicalin on MCT-induced endothelial-to-mesenchymal transition in lung

**Figure 7:**
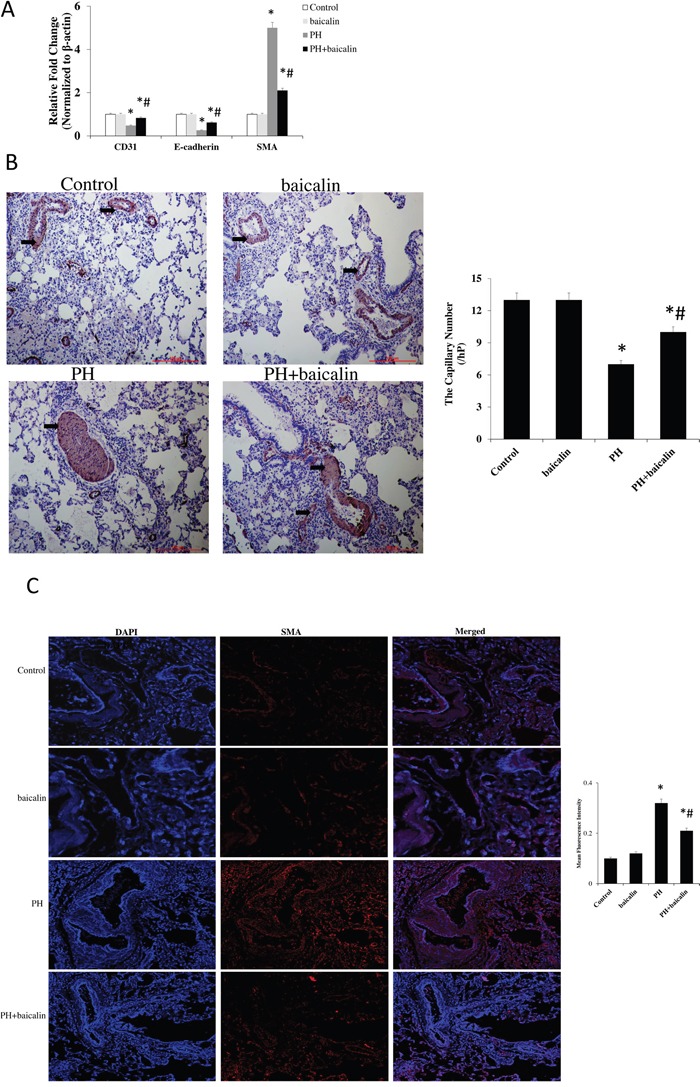
Effected of baicalin on MCT-induced endothelial-to-mesenchymal transition in lung **(A)** Quantitative real-time PCR detected the mRNA expression of pulmonary arterial endothelial cell markers CD31, and vascular endothelial cadherin E-cadherin, and α-SMA. **(B)** Immunohistochemical detected the expression of CD31. **(C)** Immunofluorescence detected the expression of α-SMA.*P<0.05, *vs*. control; ^#^*P*<0.05, *vs*. PH group.

RT-PCR exhibited a marked reduction in the pulmonary arterial endothelial cell markers CD31 and vascular endothelial cadherin (E-cadherin), however, the mesenchymal markers α-smooth muscle actin (α-SMA) was significantly increased in MCT PH rat. Furthermore, immunohistochemical andimmunofluorescence also showed a significantly decreased in the expression of CD31 but increase in the expression of α-SMA in MCT PH group compared with the control group. However, all of these changes were restored in PH + baicalin rats compared with PH rats (*P*<0.05, Figure 7).

## DISCUSSION

Our results for the first time demonstrated that intragastric administration of baicalin significantly attenuates MCT-induced pulmonary hypertension and pulmonary vascular remodeling, the protective mechanism may be through activation of BMP signaling and associated with anti-inflammatory response, induction cells apoptosis, reduction proliferation and reversal Endo-MT process. These provides the following evidences in regards to the mechanism of baicalin inhibition MCT-induced PH.

PH is a severe clinical condition associated with a poor prognosis and high mortality, characterized by narrowing and obliteration of precapillary pulmonary arteries, secondary to proliferation and apoptosis resistance of endothelial cells, smooth muscle cells, and fibroblasts. Previous reports [11, 19, 20] have demonstrated that pulmonary vascular structural remodeling of the distal pulmonary vasculature is considered to be the major pathological basis of hypoxic pulmonary hypertension, however, the exact mechanism of remodeling has not yet been fully elucidated. So far, no effective therapy is available for PH. NF-κB is a key transcriptional regulator factor, plays a key role in the process of vascular remodeling in a variety of physiological and pathophysiological states [21, 22]. Inhibition of NF-κB in the lungs can reduce the endothelial damage, attenuates the expression of infiltrating molecules, and restores the RV pressure. Previously, we demonstrated that baicalin could protect lung damage caused by MCT, inhibit the pulmonary artery pressure, reduce right ventricular hypertrophy, and attenuate pulmonary vascular remodeling, the mechanism was through inhibition of NF-κB signaling pathway related anti-inflammatory response [13], however, the further signal path mechanism is unclear.

BMPR2 mutations have been reported in more than 70% of heritable cases of PH and approximately 20% of apparently sporadic cases of idiopathic PH [23, 24]. Loss of BMPR 2 or dysfunction of BMP signaling were associated with the occurrence of PH [11]. BMPs involved in a wide range of cell function including proliferation, migration, differentiation, and apoptosis. Mounting evidence indicates that several BMPs, including BMPR2, BMP4 and BMP9 play as an important role during endocrine regulator of pulmonary arterial remodeling, cardiovascular, metabolic, and haematopoietic function [8–11]. BMPR2 signaling as a cause of increased proliferation of PASMCs playing an important role in the remodeling of pulmonary resistance vessels in PH [24], BMP9 could protect pulmonary arterial endothelial cells from apoptosis and promotes vascular stability, increase BMPR2 gene expression, and further enhancement of BMPR2 signaling.

The induction of inhibitor of DNA binding protein (Id) expression by BMP contributes to its pro-angiogenic response. Regulation of Id proteins by BMPs, with relevance to PH, play an main effect in the smooth muscle cell function [23]. Id family of transcription factors, especially Id1 and Id3 as important functional targets of BMP signaling, are potently regulated by BMP signalling in PAMSCs and might play a complementary and partially redundant role in regulating cell cycling in vascular and other tissues [25, 26]. Id family of transcription factors as important functional targets of BMP signaling, with relevance to PH, both Id1 and Id3 were downregulated in the lungs along with BMPR2 in MCT rats [27, 28]. In the present study, our data corroborated with the association of BMPs with PH, the results demonstrate the expression levels of BMP signaling related protein molecules BMP2, BMP4, BMP9, BMPR2, Id1 and Id3 were restored when the PH rats were treatment with baicalin compared with the PH rats. Reports have previously showed a direct association of NF-κB with BMP signaling in the lungs of MCT PH, inhibition of NF-κB attenuated PH and RVH by regulating BMPR2–Id axis gene in heart [12, 27]. In this report, we further confirmed that NF-κB signaling were downregulated in the PH + baicalin group than PH group. Together, our data indicate the association of NF-κB-BMP signaling with the protection of baicalin on MCT-inducced PH and pulmonary vascular remodeling.

In PH, PASMC proliferation is enhanced and apoptosis suppressed [29], previous reports have showed that a major consequence of BMPR2 mutation in PASMCs is loss of the growth suppressive effects of BMPs [30, 31]. In PAMSCs, BMPR2 mutation, leads to a proproliferative, apoptosis-resistant cell phenoype, that may contribute to the process of vascular obliteration observed in PH. In this study, we found that when the MCT rats were treatment with baicalin, the mRNA and protein expression levels of antiapoptotic gene Bcl2 was increased, but the proapoptotic genes, such as caspase-3 and Bax, was decreased in the PAH lung tissue. Importantly, our results *in vitro* also showed that baicalin could inhibit the MCT-induced proliferation and enhance apoptosis in PAMSCs. Collectively, these data suggested that the mechanism of baicalin promote apoptosis and inhibit proliferation in MCT-PH *in vivo* and *vitro* is likely to be relevant with BMP signaling. Recent studies have shown that BMP signaling is tightly regulated by inflammatory mediators/inflammation [32, 33]. Inflammatory cytokines are associated with the pathogenesis of PH, inflammatory molecules (TNF-α, IL-6 and IL-1β) were significant infiltration of macrophages and accumulation in the mice treated with MCT [34, 35]. TNF-α stimulation reduced the expression of BMP2 and BMPR-II, promote excessive PASMC proliferation and pulmonary vascular remodeling in the setting of BMPR-II deficiency in PASMCs [29]. We have previously identified the link with anti-inflammatory effects of baicalin on MCT PH rats. Mover over, our present study at least part suggested that mechanism of baicalin anti-inflammatory in PH through activation BMP signaling and inhibition pulmonary vascular remodeling in PAMSCs.

BMP antagonists, such as gremlin and noggin, are potentially important mediators of vascular changes in hypoxic pulmonary hypertension, have been implicated plays an key role in the pathophysiology of pulmonary arterial hypertension [32, 36]. Down-regulates of BMP/Smad signaling by antagonists binding directly to BMP molecules leads to a reduction in Smad1/5/8 pathway and to a concomitant increase in Smad2/3 pathway. The balance between TGF-β1 and BMP signaling also plays an important role in pulmonary fibrosis, TGF-β1 and BMP signal through a heteromeric cell surface serine/threonine kinase complex, resulting in the receptor-mediated phosphorylation and activation of Smad2/3, or Smad1/5/8 (BMPs) transcription factors and alterations in gene transcription [37]. Loss of BMPR2 function in PASMCs reduces phosphorylation of downstream Smad1/5 proteins, one of the major transcriptional targets of BMP/Smad signaling is Id protein [28]. In this report, our data showed that baicalin significantly reduced the protein expression of gremlin 1, TGF-β1, the ratio of phospho-Smad2 to total Smad2, but significantly increased the ratio of phospho-Smad 1/5/8 to total Smad 1/5/8 in the PH rats. These data provide a strong evidence for the association of BMPs with the inhibitory effect of baicalin on MCT-induced PH, the mechanistic link between BMPs/Smads axis and NF-κB during development of PH and pulmonary vascular remodeling remains unclear and warrants future investigation.

In order to further investigation the mechanism of baicalin on BMP signaling, endothelial to mesenchymal transition (Endo-MT) process was also evaluated. Endo-MT is a developmental process characterized by the acquisition of mesenchymal phenotype, such as α-SMA, and lose their surface marker protein, such as CD31 and vascular endothelial cadherin. Endo-MT has also been investigated for its potential role in vascular remodeling and the fibrotic lung disease [38–40]. Recently studies [21, 41] have showed that Endo-MT is associated with the expression of BMPR2, the dysregulation of BMPR2 signaling may initiate pulmonary endothelial cell apoptosis, Endo-MT is partially ameliorated by stimulating BMPR2 signaling even in the presence of TGF-β1. In this study, we found that a loss of vascular endothelial cadherin and CD31 and a gain of α-SMA expression in MCT-induced PH, which was reversed in baicalin rats. Taken together, we indicated that the protection of baiclin on MCT-induced PH was through suppression the Endo-MT procession and the under mechanisms was also associated with regulating BMP signaling pathways.

In conclusion, our results showed for the first time that baicalin could protection against lung damage and repair pulmonary vascular remodeling via activating BMP signaling pathways in MCT-induced PH rats *in vivo* and *vitro*. The present study indicated that bacalin possesses the abilities to anti-inflammatory response, inhibition cell proliferation and increase cell apoptosis, the underlying mechanism was associated with link BMPs/Smads axis and NF-κB signaling, which may trigger Endo-MT events in the lungs. These given new insights into the mechanisms of baicalin in MCT-indeced PH and identifies as a promising therapeutic target for PH patients.

## MATERIALS AND METHODS

### Animal protocol and cell culture

Male Wistar rats weighing 200 g to 250 g were purchased from animal experimental center of Shandong University, China. All animals received humane care in compliance with the Guide for the Care and Use of Laboratory Animals published by the U.S. National Institute of Health (NIH Publication No. 85-23, revised 1996). The study protocol was approved by the Institutional Animal Care and Use Committee (IACUC) of Shandong University, Shandong, China, and the experiments were conducted according to the Guidelines of the American Physiological Society.

The pulmonary arteries were removed from the lungs of adult rats. PASMCs were obtain from obviously report. Briefly, the cells were cultured in smooth muscle growth media (SMGM-2, Clonetics), supplemented with 5% fetal bovine serum (FBS), 1% streptomycin, and 1% penicillin. Cellular purity was >85%, as evaluated by morphological appearance under phase-contrast microscopy and immunofluorescence staining for α-actin under confocal microscopy. Cells were used between passages 3 and 6 for this study.

### Experimental protocols

Baicalin (purity >95%) was purchased from Sigma (St. Louis, MO, USA) and was dissolved in dimethyl sulfoxide (DMSO). The PH model was induced by intraperitoneal injection of 60 mg/kg MCT (Sigma-Aldrich, USA) as we described previously with modifications. Forty animals were randomly assigned to 4 groups: Control, Control+baicalin, PH and PH+baicalin groups (n=10 in each). Baicalin groups were given baicalin 100 mg/kg by intragastric administration from 2 days after MCT injection. PASMC culture were divided into 3 group according to the the cells were obtained from different animals: control, PH and baicalin group. Cell viability was analyzed by 3-(4, 5-Dimethylthiazol-2-yl)-2, 5-diphenyltetrazolium Bromide (MTT). Cell proliferation was assessed using a DNA BrdU incorporation assay (Roche Applied Science, Burgess Hill, UK). Briefly, cells were seeded into 96-well cell culture plates at a density of 1×10^4^ cells. BrdU was incorporated into proliferating cells according to the manufacturer's protocol. The absorbance of the plate was measured by a spectrophotometer microplate reader at a dual wavelength of 450/550 nm.

### Determination of PH model

30 days after MCT injection, the experimental rats were anesthetized with pentobarbital (30 mg/kg, ip, Sigma Aldrich) and inserted with a 3F-Miller micro tip catheter via the right jugular vein into the right ventricle to obtain the right ventricular systolic pressure (RVSP). Results of the hemodynamic parameters, right ventricular hypertrophy, and pulmonary arterial pathological changes were used to evaluate whether the model of pulmonary arterial hypertension was successfully established. The ratio of right ventricular weight to left ventricle plus septum (RV/LV + S) was calculated to measure right ventricular hypertrophy.

### Immunological and immunohistochemical analyses

The rats were sacrificed after hemodynamic measurements, and the lung and heart were quickly harvested and fixed *in situ* via the trachea cannula with buffered 4% formaldehyde, and embedded in paraffin. The sections were cut into 4-5 μm slices and were stained with streptavidin peroxidase and hematoxylin and eosin (H&E). For immunohistochemistry, 4-5 μm-thick cryosections or PAMSCs were first blocked with 5% goat serum (ab7481; Abcam, Cambridge, UK) for 30 min. The sections were then incubated with proliferating cell nuclear antigen (PCNA) (ab18197, Abcam) and CD31 (ab28364; Abcam). Subsequently, the 3, 3′- diaminobenzidine (DAB) dye was added to visualize the antibodies, and following washing of the tissue sections with phosphate-buffered saline (PBS) solution, the sections were observed and photographed under a microscope. For immunofluorescence, the sections or cells were incubated with rabbit polyclonal to smooth muscle α-actin (α-SMA, ab5694) or a nonspecific IgG antibody for 1 h at room temperature, which was followed by 1-h incubation in the dark with florescence isothiocyanate–conjugated goat anti-rabbit secondary antibody (ZF-0311; ZSGB-Bio Co., Beijing, China). Fluorescent images were taken with a Nikon Eclipse 90i microscope. Cells were fixed and permeabilized in a 50%.

### RNA extraction and quantitative real-time polymerase chain reaction

Total RNA was extracted using RNeasy kit (Qiagen, Valencia, CA) and reverse transcription was performed using iScript cDNA synthesis kit (Bio-Rad, Hercules, CA) according to the manufacturer's instructions. Quantitative real-time polymerase chain reaction(qRT-PCR) analysis was performed to detect the relative pulmonary expression levels of BMP2, BMP4, BMP9, BMPR2, Id1, Id3, Bax, Bcl2, caspase-3 CD31, α-smooth muscle actin (α-SMA), E-cadherin and TNF-α, IL-6, and IL-1β using gene-specific primers as described previously.

### Western blot

Lung tissue were pulverized in liquid nitrogen and cytosolic, and nuclear proteins were extracted using NE-PER nuclear and cytosolic extraction reagents (Pierce). Protein extraction buffer and equal amounts of protein were denatured and separated by sodium dodecyl sulfatepolyacryl-amide gel electrophoresis (SDS-PAGE). Protein concentrations were assessed using the BCA Protein Assay kit (Santa Cruz Biotechnology). 10 μg of total protein were electrophoresed on 4-20% gradient SDS-PAGE gels and transferred to a nitrocellulose membrane. Western blotting and the subsequent quantification of each blots was performed, as described previously. The primary antibodies used in this study include BMPR2 (ab170206), IκB-α (ab76429), NF-κB p65(ab16502), p-NF-κBp65 (ab86299), BMP4 (ab39973), BMP9 (ab35088), gremlin (sc-18274), transforming growth factor-β1 (TGF-β1; ab25121), Smad2/3 (sc-133098), p-Smad2/3 (sc-11769), Smad1/5/8 (sc-6031), p-Smad1/5/8 (sc-12353), Bax (ab32503), Bcl2 (ab59348), cleaved caspase-3 (ab13847), Histone H3 (ab8898) and GAPDH (ab181602).

### Flow cytometry

The Annexin V-FITC/PI apoptosis detection kit according to the manufacturer's instructions (Roche Diagnostics, Indianapolis, IN, USA). Briefly, PAMSCs were seeded in 6-well plates. 1×10^6^ cells were collected and suspended in 500 μl binding buffer, and 5 μl Annexin V-FITC and 5 μl PI were added to each sample and incubated in the dark for 15 min. The cell surface phosphatidylserine in apoptotic cells was quantitatively estimated by using Annexin V/FITC apoptosis detection kit according to manufacturer's instructions (Roche Diagnostics).

### Terminal deoxynucleotidyl transferase-mediated deoxyuridine triphosphate nick-end

Apoptosis was detected using a One Step TUNEL Apoptosis Assay Kit (Beyotime Institute of Biotechnology, Shanghai, China) according to protocol. Briefly, cells were seeded on coverslips in 24-well plates at a density of cells for 24 h. After fixing with 4% formaldehyde, the cells were washed with PBS and permeabilized using 0.2% Triton X-100. Following equilibration, cells were labeled using TdT reaction mix and incubated for 1 h at 37°C in a humidified chamber. Subsequently, 2× SSC were added for 15 min to stop the reaction. Apoptotic cells were detected using a Nikon Eclipse 90i microscope (Nikon Corporation; Tokyo, Japan).

### Statistical analysis

All experiments were performed at least three times for each determination. Data are expressed as mean ± SD. Comparisons of parameters between 2 groups were made with unpaired Student t test. Comparisons of parameters among 3 groups were made with one-way analysis of variance (ANOVA), followed by the Scheffe multiple-comparison test. Statistical analysis was carried out by using the SPSS 13.0 software. *P* <0.05 was regarded as significant statistical difference.
